# Identification and Transcriptomic Analysis of Mitochondria-Related Gene Signatures in Obesity

**DOI:** 10.3390/metabo16060419

**Published:** 2026-06-15

**Authors:** Hezhang Yun, Chang Liu, Binghong Gao, Peijie Chen

**Affiliations:** 1 Faculty of Health Sciences and Sports, Macao Polytechnic University, Macao, China; runhzdyx@bsu.edu.cn; 2 School of Exercise and Health, Shanghai University of Sport, Shanghai 200438, China; 3 School of Sport Science, Beijing Sport University, Beijing 100084, China; 4 School of Athletic Performance, Shanghai University of Sport, Shanghai 200438, China

**Keywords:** obesity, mitochondrial dysfunction, bioinformatics, machine learning, molecular docking, immune infiltration

## Abstract

**Objectives**: This study aimed to identify core genes associated with mitochondria-related transcriptomic signatures and evaluate their potential as computational biomarkers, immune characteristics, regulatory mechanisms, and potential therapeutic relevance. **Methods**: Obesity-related transcriptome datasets were obtained from the GEO database. Differentially expressed genes (DEGs) were intersected with mitochondria-related genes (MRGs) to identify obesity-related MRGs. Functional enrichment, protein–protein interaction (PPI) analysis, CytoHubba, LASSO and random forest algorithms were used to screen core genes. External validation, ROC analysis, immune infiltration analysis, regulatory network construction, candidate drug prediction, and molecular docking were further performed. **Results**: A total of 527 DEGs and 15 differentially expressed MRGs were identified. Enrichment analysis suggested that these mitochondria-related genes were mainly associated with disrupted mitochondrial energy metabolism, lipid metabolic remodeling, and altered substrate utilization. *ECHDC2*, *FASN*, *NAT8L*, and *AASS* were identified as core MRGs; these genes are respectively associated with mitochondrial metabolic regulation, de novo fatty acid synthesis, N-acetylaspartate-related mitochondrial metabolism, and lysine degradation. These genes were significantly downregulated in obesity and showed good diagnostic performance. Immune infiltration analysis revealed alterations in the immune microenvironment, and the core genes were negatively correlated with multiple immune cell types. Molecular docking showed that Genistein had the lowest predicted binding free energy with NAT8L (−8.89 kcal/mol), suggesting relatively favorable binding among the tested ligand–target pairs. **Conclusions**: *ECHDC2*, *FASN*, *NAT8L*, and *AASS* may serve as candidate computational biomarkers, among which *FASN* represents a known lipid metabolism-related gene, supporting the biological plausibility of the workflow.

## 1. Introduction

Obesity is a chronic and complex disease driven by multiple interacting factors and has become a major global public health concern [1]. According to the World Health Organization, more than 1 billion people worldwide were living with obesity in 2022, and the prevalence of obesity has more than doubled among adults and quadrupled among children and adolescents since 1990 [2]. Obesity is characterized not only by excessive accumulation of body fat but also by its close association with the development and progression of type 2 diabetes mellitus, cardiovascular diseases, non-alcoholic fatty liver disease, and various other chronic disorders [3,4,5,6]. Studies have shown that obesity is not simply caused by an imbalance between energy intake and expenditure but represents a systemic pathological process involving adipose tissue dysfunction, chronic low-grade inflammation, and abnormal inter-organ signaling [7,8,9]. Under obese conditions, adipocyte hypertrophy, dysregulated lipid storage, and abnormal immune cell infiltration interact with one another, jointly contributing to the development of insulin resistance and systemic metabolic disorders.

Mitochondria play a critical role in eukaryotic cells [10,11]. Increasing evidence suggests that mitochondrial dysfunction is one of the key pathological bases of obesity and its related metabolic abnormalities. In obesity, adipose tissue and its associated cells may exhibit impaired mitochondrial fatty acid metabolism, increased accumulation of reactive oxygen species, and defective mitochondrial biogenesis, thereby affecting adipocyte differentiation, lipid metabolism, thermogenic capacity, and insulin sensitivity [12,13]. In addition, mitochondrial abnormalities can contribute to the formation and maintenance of the chronic inflammatory microenvironment associated with obesity by regulating redox homeostasis, metabolic intermediates, and inflammatory signaling pathways [14,15]. These findings suggest that mitochondrial dysfunction may represent an important mechanistic link between metabolic imbalance and immune dysregulation. Unlike previous obesity-related bioinformatics studies that primarily focused on global differentially expressed genes or general metabolic/inflammatory signatures, the present study specifically integrated obesity-related transcriptomic profiles with a curated mitochondria-related gene set to prioritize candidate MRGs associated with mitochondria-related transcriptomic signatures in subcutaneous adipose tissue.

Although mitochondrial dysfunction has been increasingly recognized as an important contributor to obesity-related metabolic abnormalities, the key mitochondria-related genes and regulatory networks involved in this process remain incompletely characterized. In particular, it remains unclear which mitochondria-related genes are consistently dysregulated in obese adipose tissue, whether these genes can distinguish obese individuals from normal-weight individuals at the transcriptomic level, and how they are associated with immune microenvironment remodeling and potential therapeutic regulation. We hypothesized that specific mitochondria-related genes are differentially expressed in obese adipose tissue and may serve as computational biomarkers reflecting mitochondria-related transcriptomic signatures. Based on this hypothesis, the present study integrated obesity-related transcriptomic datasets from the GEO database with curated mitochondria-related gene sets to identify core MRGs and further evaluate their functional characteristics, immune associations, regulatory networks, and potential therapeutic relevance.

## 2. Materials and Methods

### 2.1. Acquisition and Preprocessing of Microarray Datasets

The obesity-related gene expression profile data used in this study were obtained from the Gene Expression Omnibus (GEO) database. Three datasets were included: GSE94752, GSE55200, and GSE151839. Among them, GSE94752 and GSE55200 were used as the training datasets, whereas GSE151839 was used as an external validation dataset. The platform information, tissue sources, and applications of each dataset are shown in Table 1 and Supplementary Table S1.

Raw microarray data were preprocessed, which performed background correction, quantile normalization and probe-level summarization in one step, after which probe signals were converted into gene expression values. Considering the technical variations caused by different experimental batches, batch effects were corrected using the removeBatchEffect function in the limma package. The corrected expression matrix was then used for subsequent differential expression analysis and machine learning modeling. Therefore, the final DEG list used for downstream analyses was not generated by directly taking the union or intersection of two independently derived DEG lists but by applying a unified differential expression analysis to the merged and batch-corrected training cohort. To evaluate the effectiveness of batch effect correction, PCA analysis was performed before and after correction, and the expression consistency of five commonly detected housekeeping genes, including *ACTB*, *HPRT1*, *RPLP0*, *TBP*, and *PPIA*, was further assessed between the two training datasets. The corresponding PCA plots and housekeeping gene consistency results are shown in Supplementary Figure S1 and Supplementary Table S2, respectively.

### 2.2. Inclusion and Exclusion Criteria

Datasets were included if they met the following criteria: (1) human adipose tissue transcriptomic datasets related to obesity; (2) samples with clearly annotated obese and normal-weight or lean control groups; (3) availability of raw or normalized gene expression data suitable for differential expression analysis; (4) datasets generated from subcutaneous adipose tissue; and (5) datasets with sufficient gene annotation information for mapping probes to gene symbols.

Datasets were excluded if they met any of the following criteria: (1) non-human datasets or animal-/cell-line-only studies; (2) datasets without clearly defined obese and control groups; (3) datasets derived from tissues other than subcutaneous adipose tissue; (4) datasets lacking accessible expression matrices or adequate annotation information; (5) duplicate datasets or datasets with overlapping samples; and (6) datasets in which samples had insufficient clinical or grouping information for classification into OB and NW groups.

### 2.3. Identification of Differentially Expressed Genes

Differential expression analysis between the obese group and healthy control group in the training dataset was performed using the limma R package, followed by Bonferroni correction. Genes with an adjusted *p* value < 0.05 and |log_2_FC| > 0.5 were considered DEGs. Volcano plots were generated using ggplot2, and heatmaps of the most significantly differentially expressed genes were constructed using ComplexHeatmap.

### 2.4. Identification of Mitochondria-Related Genes

The human mitochondria-related gene set was obtained from the MitoCarta3.0 database, which is a widely used resource for mitochondrial protein and pathway annotation and contains 1136 human MRGs [16]. The DEGs were intersected with the mitochondria-related gene set from MitoCarta3.0 to identify obesity-related differentially expressed MRGs. Venn diagrams were generated using R software (version 4.2.1) to visualize the overlapping genes.

### 2.5. Functional Enrichment Analysis

To further investigate the potential biological functions and signaling pathways of differentially expressed MRGs, enrichment analyses were performed using the clusterProfiler package in R software (version 4.2.1). Gene Ontology (GO) analysis included three categories: biological process (BP), molecular function (MF), and cellular component (CC). In addition, the Kyoto Encyclopedia of Genes and Genomes (KEGG) database was used to identify relevant metabolic pathways. A significance threshold of *p* < 0.05 was applied.

### 2.6. Construction of the Protein–Protein Interaction Network

The identified differentially expressed MRGs were imported into the STRING database for PPI analysis [17], with the minimum required interaction score set to 0.15. The results were then imported into Cytoscape 3.10.4 for network visualization. In the network, node color intensity was set according to degree values, with darker nodes indicating higher degree values. Hub genes were identified using the CytoHubba plugin in Cytoscape. Five topological algorithms, including Maximum Neighborhood Component (MNC), Degree, Edge Percolated Component (EPC), Density of Maximum Neighborhood Component (DMNC), and Maximal Clique Centrality (MCC), were used to screen the top 10 candidate genes, respectively. The intersection of genes identified by these five algorithms was defined as the hub genes in the PPI network.

### 2.7. Machine Learning-Based Screening of Feature Genes

To identify a robust set of diagnostic feature genes, machine learning methods were applied to screen differentially expressed MRGs: least absolute shrinkage and selection operator (LASSO) regression and random forest (RF). Candidate genes obtained from the algorithms were intersected to reduce the risk of model overfitting and improve the robustness of feature selection.

#### 2.7.1. LASSO Regression Analysis

The glmnet R package was used to construct the LASSO regression model. Sample grouping, namely obesity or healthy control, was used as the dependent variable, and the MRG expression matrix was used as the independent variable. Ten-fold cross-validation was performed to determine the optimal penalty parameter λ, and lambda.min, corresponding to the minimum cross-validation error, was selected as the final parameter. Genes with non-zero regression coefficients were defined as candidate feature genes identified by LASSO regression.

#### 2.7.2. Random Forest Analysis

A random forest classification model was constructed using the randomForest R package, with the number of decision trees set to 500. Genes were ranked according to variable importance scores, and candidate feature genes with high importance were selected. The intersection of genes identified by LASSO and RF was defined as the machine learning-derived candidate key genes. A Venn diagram was used to display the overlap among the results obtained from the algorithms.

### 2.8. Determination of Hub MRGs

To prioritize candidate mitochondria-related genes with both network topological relevance and transcriptome-based classification potential, genes selected by the machine learning algorithms were intersected with hub genes derived from the PPI network. This integrative strategy was used for candidate gene prioritization rather than independent validation. The resulting genes were defined as prioritized core MRGs and were subsequently evaluated in an external validation dataset. Correlation analysis among the core genes was performed based on their expression matrices, and the correlation heatmaps were visualized in the training and validation datasets.

### 2.9. Evaluation of the Diagnostic Performance of Key Biomarkers

In the combined training dataset, receiver operating characteristic (ROC) curves were generated using the pROC R package, and the area under the curve (AUC) was calculated to evaluate the diagnostic performance of each hub MRG as a single-gene biomarker for obesity. An AUC value > 0.70 is generally considered to indicate good discriminative ability [18,19]. The GSE151839 dataset was then used as an external validation cohort to verify the expression differences and diagnostic performance of the hub MRGs in an independent cohort. Graphical visualization was performed using ggplot2.

### 2.10. Immune Cell Infiltration Analysis

Single-sample gene set enrichment analysis (ssGSEA) was used to evaluate immune cell-associated transcriptomic enrichment patterns in each sample. ssGSEA calculates an enrichment score for each predefined gene set in each individual sample and has been used to characterize pathway- or cell-type-associated transcriptomic signatures in bulk expression data [20]. In addition, xCell and related gene-signature-based approaches have demonstrated the feasibility of inferring immune- and stromal cell-type-associated signals from bulk gene expression profiles [21]. Previous work on human adipose tissue further showed that cell-type-enriched transcriptomic signatures can be resolved from unfractionated adipose tissue RNA-seq datasets [22]. 

Therefore, ssGSEA was used in the present study to estimate relative immune cell-associated transcriptional signatures rather than direct immune cell abundance. Therefore, ssGSEA was used in the present study to estimate relative immune cell-associated transcriptional signatures rather than direct immune cell abundance. Immune-related enrichment scores and infiltration distributions were visualized using the ggplot2 package (version 3.4.4). Intergroup statistical comparisons were performed using the stats package (version 4.2.1) and car package (version 3.1-0), and a *p* value < 0.05 was considered statistically significant.

### 2.11. Construction of the Transcription Factor–mRNA–miRNA Regulatory Network

To explore the potential upstream regulatory mechanisms of the hub MRGs, a transcription factor (TF)–mRNA–miRNA regulatory network was constructed using the NetworkAnalyst platform (https://www.networkanalyst.ca/) [23]. TF–gene interaction information was obtained from the JASPAR database, whereas miRNA–target gene interaction information was obtained from the miRTarBase database. The resulting regulatory relationships were imported into Cytoscape for network integration and visualization.

### 2.12. Screening of Potential Drugs

Potential small-molecule drugs targeting the hub MRGs were predicted using the Drug Signatures Database (DSigDB) module of the Enrichr online platform. Candidate compounds were screened according to enrichment significance, and a drug–target interaction network was constructed by integrating the corresponding target gene information.

### 2.13. Molecular Docking Analysis of Predicted Drugs and Target Proteins

To further evaluate the binding potential between candidate drugs and key target proteins, the three-dimensional structures of target proteins were obtained from the Protein Data Bank (PDB) database, and the structures of small-molecule ligands were downloaded from the PubChem database. For proteins without suitable experimentally resolved human structures, AlphaFold-predicted models were used. The structural information of target proteins used or considered in the molecular docking analysis, including structure source, structure type or coverage, mean pLDDT score, Ramachandran plot results, and docking usage, is provided in Supplementary Table S3. For AASS, PDB 5L78 was listed only as available domain-level structural information because it corresponds to the saccharopine dehydrogenase domain and may not represent the full-length AASS protein. Since no candidate compound was predicted to directly target AASS in the DSigDB analysis, AASS was not included in the final docking analysis.

Receptors and ligands were preprocessed using AutoDock Tools (version 1.5.7), including the removal of water molecules, addition of polar hydrogen atoms, and assignment of Gasteiger charges. Molecular docking simulations were performed using AutoDock Vina version 1.2.7, and binding affinity between ligands and receptors was evaluated based on binding free energy values expressed in kcal/mol [24]. The docking parameters were set as follows: exhaustiveness = 32, num_modes = 9, and energy_range = 3 kcal/mol. For each receptor protein, the grid box was individually defined to encompass the entire protein structure. Finally, docking results were visualized using PyMOL (version 2.6).

## 3. Results

### 3.1. Data Preprocessing and Identification of Differentially Expressed Genes

**Figure 1:**
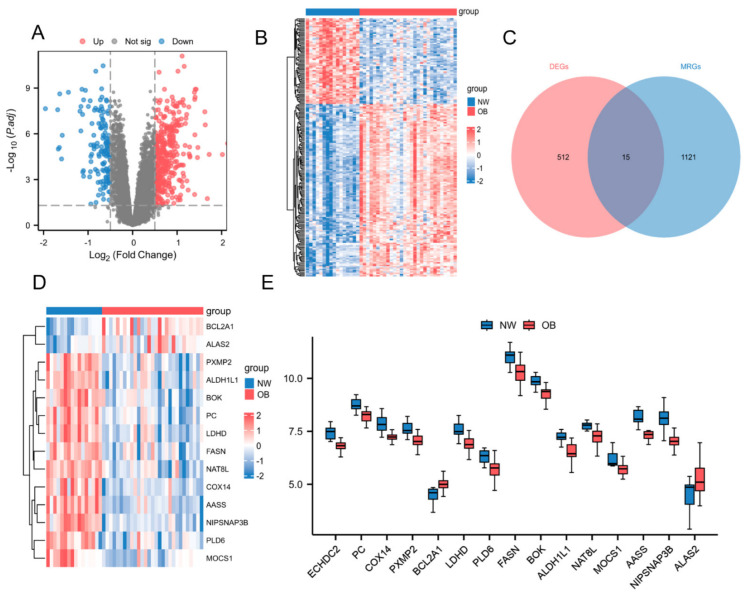
Differential expression analysis. (**A**) Volcano plot of DEGs between obese and normal samples, dashed vertical lines indicate the log_2_FC thresholds of −0.5 and 0.5, and the dashed horizontal line indicates the adjusted *p* value threshold of 0.05; (**B**) heatmap of the top 200 DEGs; (**C**) Venn diagram showing 15 differentially expressed mitochondria-related genes; (**D**) heatmap of the 15 overlapping MRGs; (**E**) boxplots of the 15 overlapping MRGs; OB, obesity group; NW, normal-weight healthy control group.

In this study, expression matrices from two GEO datasets were included, comprising 29 obese patients and 16 healthy controls. After batch effect correction, a total of 527 DEGs were identified, including 386 upregulated and 141 downregulated genes. The volcano plot is shown in Figure 1A and Supplementary Table S4. The top 200 most significant DEGs were further selected to generate a heatmap, as shown in Figure 1B.

To explore the relationship between obesity and mitochondrial dysfunction, the DEGs were intersected with MRGs, resulting in 15 overlapping differentially expressed MRGs (Figure 1C). Subsequently, heatmap analysis (Figure 1D) and boxplot analysis (Figure 1E) were performed to illustrate the expression differences in these 15 MRGs between the obese and control groups. The results showed distinct expression profiles of these genes between obese and normal tissues, suggesting that their dysregulated expression may play an important role in the occurrence and progression of obesity.

### 3.2. Identification and Functional Enrichment Analysis of Differentially Expressed MRGs

**Figure 2:**
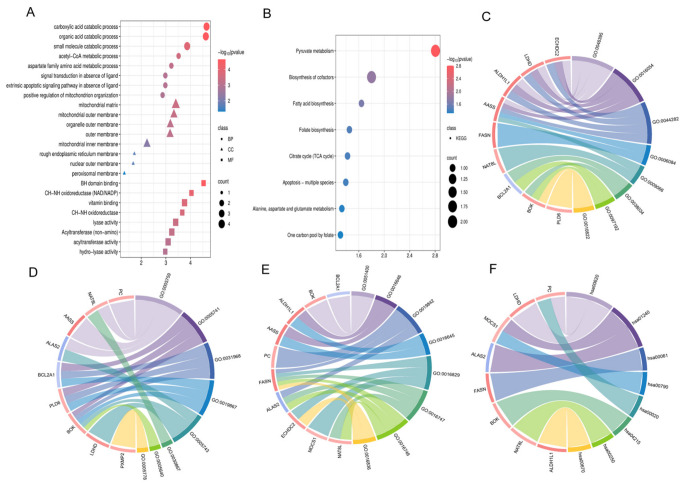
Functional enrichment analysis of key genes. (**A**) GO enrichment analysis results; (**B**) KEGG pathway enrichment analysis results; (**C**) chord diagram showing the association between key genes and BP terms; (**D**) chord diagram showing the association between key genes and CC terms; (**E**) chord diagram showing the association between key genes and MF terms; (**F**) chord diagram showing the association between key genes and KEGG pathways.

Functional enrichment analysis of the differentially expressed MRGs was performed using R software. GO analysis showed that these genes were mainly enriched in BPs such as the carboxylic acid catabolic process, small-molecule catabolic process, and acetyl-CoA/acyl-CoA metabolic processes. In terms of CC, these genes were primarily localized to the mitochondrial matrix, mitochondrial inner membrane, and mitochondrial outer membrane. Regarding MF, they were mainly involved in oxidoreductase activity, acetyltransferase/acyltransferase activity, and vitamin binding (Figure 2A).

KEGG pathway analysis showed that these genes were mainly enriched in pathways related to energy metabolism and coenzyme biosynthesis, including pyruvate metabolism, the citrate cycle (TCA cycle), fatty acid biosynthesis, alanine, aspartate and glutamate metabolism, biosynthesis of cofactors, folate biosynthesis, one-carbon pool by folate, and apoptosis in multiple species (Figure 2B). In addition, four chord diagrams (Figure 2C–F) further illustrated the relationships between genes and GO/KEGG functional terms, as well as the overlapping enrichment of shared genes among different functional categories. These results intuitively reflected the multifaceted roles of MRGs in obesity-related metabolic regulation and mitochondrial function maintenance.

### 3.3. Construction of the PPI Network and Screening of Hub Genes

**Figure 3:**
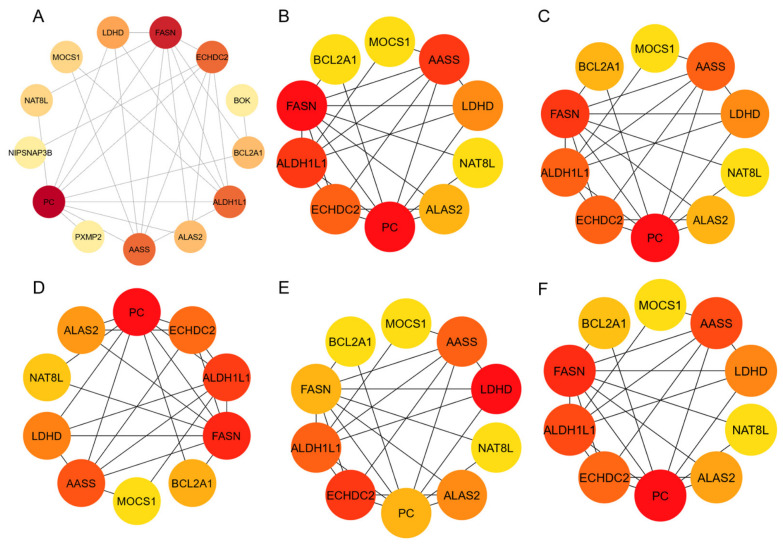
Construction of the PPI network and screening of hub genes among MRGs. (**A**) PPI network constructed using the STRING database, node color intensity represent degree values; darker nodes indicate higher degree values; (**B**) screening results based on the MNC algorithm; (**C**) screening results based on the Degree algorithm; (**D**) screening results based on the EPC algorithm; (**E**) screening results based on the DMNC algorithm; (**F**) screening results based on the MCC algorithm.

**Figure 4:**
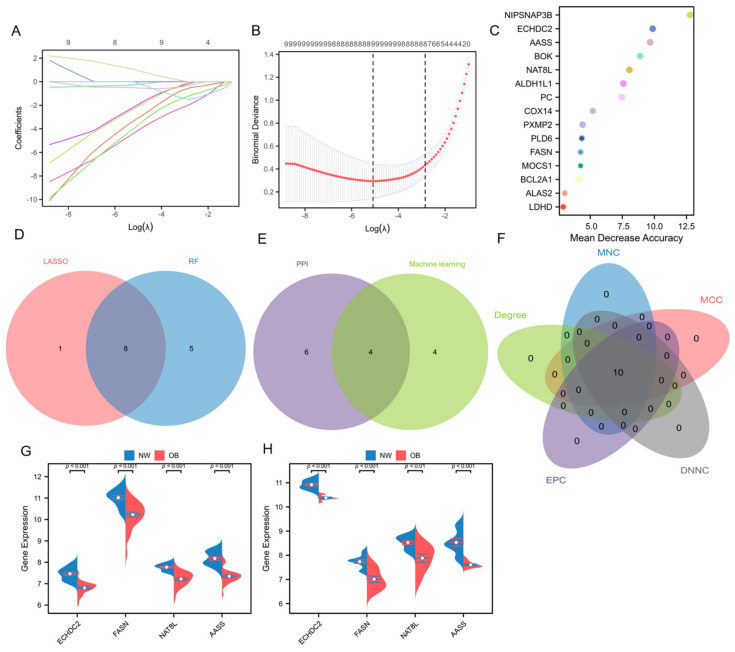
Identification of feature MRGs using machine learning algorithms. (**A**) Determination of the optimal parameter lambda in the LASSO model; (**B**) coefficient distribution of related MRGs based on the optimal lambda; (**C**) screening of feature MRGs using random forest; (**D**) Venn diagram showing the overlapping genes identified by the machine learning algorithms; (**E**) key MRGs identified by integrating machine learning algorithms and CytoHubba analysis; (**F**) hub genes obtained from the intersection of five algorithms; (**G**) violin plots showing the expression of key MRGs in the training dataset. The exact *p* values for the comparison as follows: ECHDC2, *p* = 3.6 × 10^−8^; FASN, *p* = 6.7 × 10^−6^; NAT8L, *p* = 3.7 × 10^−9^; and AASS, *p* = 4.4 × 10^−9^; (**H**) violin plots showing the expression of key MRGs in the validation dataset. The exact p values for the comparison as follows: ECHDC2, *p* = 1.2 × 10^−6^; FASN, *p* = 2.8 × 10^−4^; NAT8L, *p* = 5.0 × 10^−3^; and AASS, *p* = 1.6 × 10^−4^; OB, obesity group; NW, normal-weight healthy control group.

To clarify the interactions among proteins encoded by the MRGs, a PPI network was constructed based on the STRING database. The final PPI network contained 13 nodes and 26 edges (Figure 3A). Subsequently, the CytoHubba plugin in Cytoscape was used to screen key genes using five algorithms, namely MNC, Degree, EPC, DMNC, and MCC. The top 10 genes ranked by each algorithm were extracted to construct significant modules (Figure 3B–F and Supplementary Table S5). After intersection analysis of the results obtained from the five algorithms, 10 hub genes were ultimately identified: *PC*, *FASN*, *ALAS2*, *MOCS1*, *AASS*, *NAT8L*, *ALDH1L1*, *ECHDC2*, *LDHD*, and *BCL2A1* (Figure 4F).

### 3.4. Machine Learning-Based Screening of Candidate Feature Genes

To further identify potential biomarkers for obesity, machine learning algorithms were applied for feature gene screening. LASSO regression identified 9 candidate genes (Figure 4A,B), and RF identified 13 candidate genes (Figure 4C). After intersecting the results obtained from the algorithms, eight key genes were identified: *ECHDC2*, *COX14*, *PLD6*, *FASN*, *NAT8L*, *BOK*, *NIPSNAP3B* and *AASS* (Figure 4D; Supplementary Table S6).

### 3.5. Integrated Screening of Core MRGs

The key genes identified by the machine learning algorithms were further intersected with the hub genes identified from the PPI network. Finally, four core MRGs were identified: *ECHDC2*, *FASN*, *NAT8L*, and *AASS* (Figure 4E). In both the training dataset (Figure 4G) and the validation dataset (Figure 4H), the expression levels of these four core MRGs were significantly lower in the OB group than in the NW group, with statistically significant differences Supplementary Table S7. To further evaluate the relationships among the four core MRGs, correlation heatmaps were generated in both the training and validation datasets (Supplementary Figure S2). 

### 3.6. Evaluation of Transcriptome-Based Discriminatory Performance in Training and External Validation Datasets

**Figure 5:**
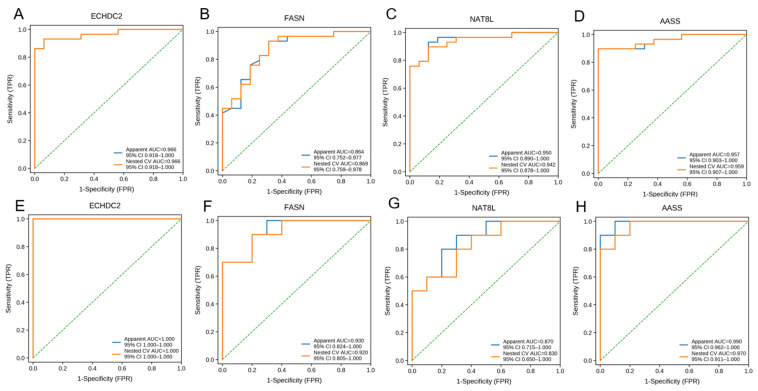
Validation of computational biomarkers. (**A**) ROC curve of ECHDC2 in the training dataset; (**B**) ROC curve of FASN in the training dataset; (**C**) ROC curve of NAT8L in the training dataset; (**D**) ROC curve of AASS in the training dataset; (**E**) ROC curve of ECHDC2 in the validation dataset; (**F**) ROC curve of FASN in the validation dataset; (**G**) ROC curve of NAT8L in the validation dataset; (**H**) ROC curve of AASS in the validation dataset.

The results indicated that the diagnostic model based on the four core MRGs could effectively distinguish OB patients from NW individuals. In the training dataset (Figure 5A–D), the areas under the ROC curve (AUCs) for *ECHDC2*, *FASN*, *NAT8L*, and *AASS* were 0.966, 0.864, 0.950, and 0.957, respectively, with corresponding 95% confidence intervals (CIs) of 0.918–1.000, 0.752–0.977, 0.890–1.000, and 0.903–1.000.

The diagnostic performance of these genes was further validated in an independent validation dataset. As shown in Figure 5E–H, the AUCs of *ECHDC2*, *FASN*, *NAT8L*, and *AASS* were 1.000, 0.930, 0.870, and 0.990, respectively, with corresponding 95% CIs of 1.000–1.000, 0.824–1.000, 0.715–1.000, and 0.962–1.000. These results indicate that the expression levels of the four prioritized genes showed discriminatory potential between OB and NW samples within the analyzed transcriptomic datasets. However, these findings should not be interpreted as evidence of established clinical diagnostic performance without validation in larger independent cohorts and experimental studies.

To obtain a more conservative estimate of model performance, nested stratified k-fold cross-validation was performed using an outer 5-fold and inner 3-fold framework. The nested cross-validated AUCs in the training dataset were 0.966, 0.869, 0.942, and 0.959 for *ECHDC2*, *FASN*, *NAT8L*, and *AASS*, respectively, whereas those in the validation dataset were 1.000, 0.920, 0.830, and 0.970, respectively.

### 3.7. Construction of the miRNA–MRG–TF Regulatory Network

**Figure 6:**
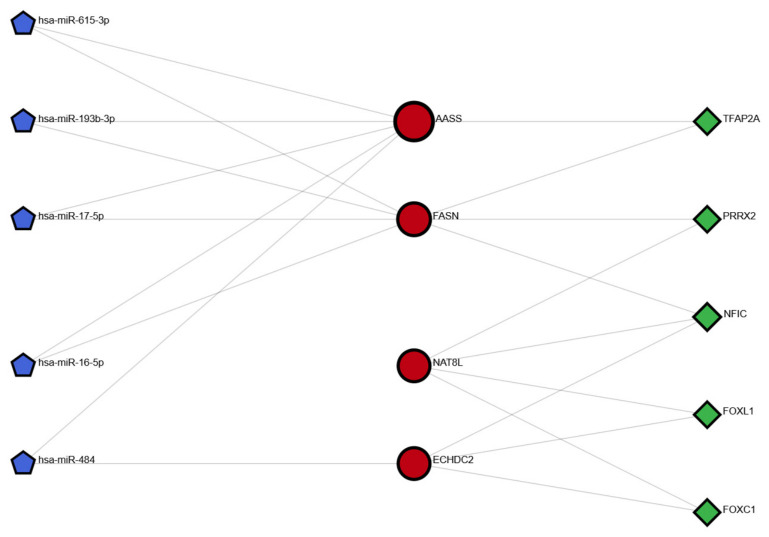
miRNA–MRG–TF regulatory network constructed.

An miRNA–mRNA regulatory network was constructed based on the miRTarBase database to predict the potential upstream miRNAs of *ECHDC2*, *FASN*, *NAT8L*, and *AASS*. A total of 71 miRNAs were retrieved, among which hsa-miR-16-5p, hsa-miR-17-5p, hsa-miR-484, hsa-miR-193b-3p, and hsa-miR-615-3p were predicted to regulate multiple target genes simultaneously. To improve figure readability, the simplified miRNA–mRNA–TF regulatory network is now presented as Figure 6 in the main manuscript, whereas the full regulatory network has been moved to Supplementary Figure S3.

In addition, 26 transcription factors (TFs) were predicted using the NetworkAnalyst platform, among which NFIC, PRRX2, FOXC1, FOXL1, and TFAP2A were able to regulate multiple core genes. The final miRNA–mRNA–TF regulatory network consisted of 101 nodes and 108 edges. Because the network had a relatively low average degree, most regulatory nodes were connected to only one or a few target genes. This sparse topology suggests that the predicted upstream regulatory relationships should be interpreted cautiously and regarded as hypothesis-generating rather than definitive regulatory evidence.

### 3.8. Results of Immune Cell Infiltration Analysis

**Figure 7:**
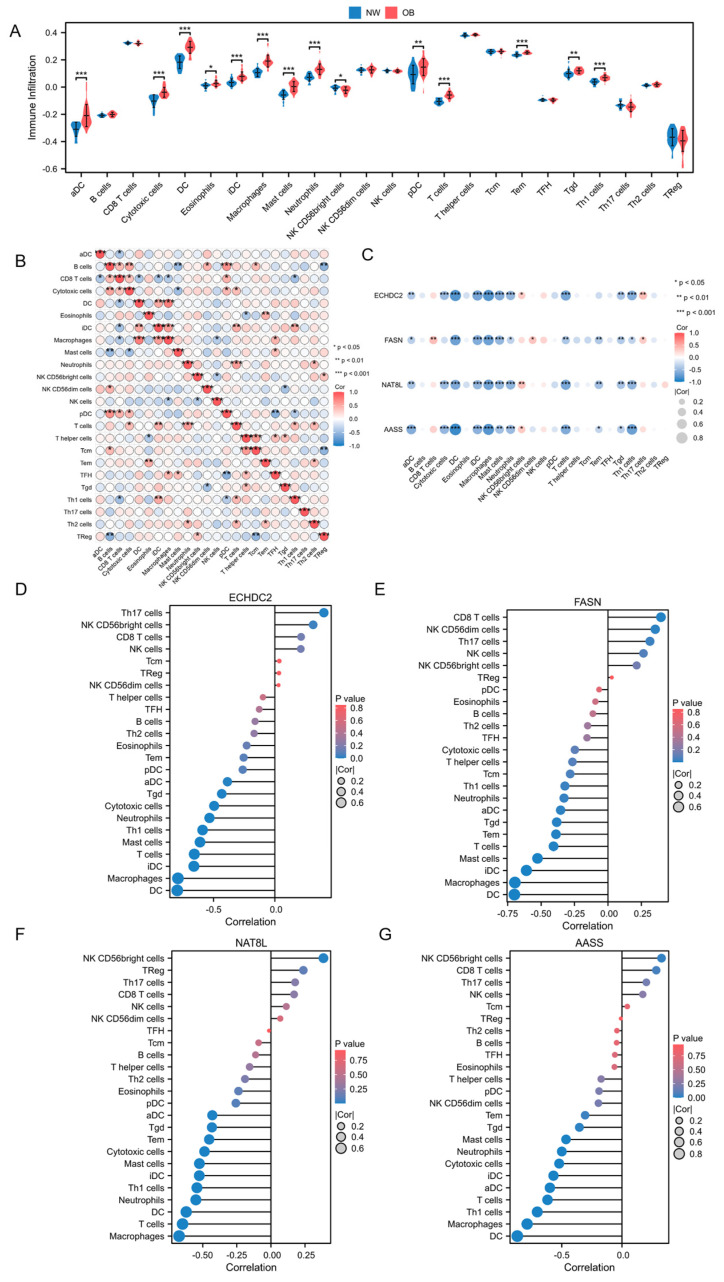
Immune status analysis based on the four key MRGs. (**A**) Violin plots showing differences in immune cell infiltration between the OB and NW groups; (**B**) heatmap showing correlations among immune cell subsets; (**C**) correlations between the four key MRGs and immune cells; (**D**) lollipop plot showing correlations between ECHDC2 and immune cells; (**E**) lollipop plot showing correlations between FASN and immune cells; (**F**) lollipop plot showing correlations between NAT8L and immune cells; (**G**) lollipop plot showing correlations between AASS and immune cells. * *p* < 0.05, ** *p* < 0.01, *** *p* < 0.001; OB, obesity group; NW, normal-weight healthy control group.

The results showed marked differences in immune-related gene signatures in the OB group (Figure 7A). Overall, compared with the NW group, most immune cell-associated signatures showed an increasing trend in the OB group, suggesting that obesity may be accompanied by remodeling of the immune microenvironment in subcutaneous adipose tissue. However, because ssGSEA infers relative enrichment scores based on predefined immune-related gene sets, these results should be interpreted as changes in immune cell-associated transcriptomic signatures rather than direct measurements of immune cell abundance.

### 3.9. Correlation Analysis Between Immune Cell Subsets and MRGs

Pairwise correlation analysis of 24 immune cell subsets was performed to systematically reveal the synergistic and antagonistic relationships among immune cells in the immune microenvironment associated with mitochondria-related transcriptomic signatures. As shown in Figure 7B, CD8 T cells were negatively correlated with aDCs, DCs, iDCs, and macrophages but positively correlated with B cells, cytotoxic cells, and pDCs. B cells were positively correlated with NK CD56dim cells and Tcm cells. Macrophages were positively correlated with DCs and iDCs, indicating significant associations among multiple immune cell subsets.

Further correlation analysis between the four core MRGs and immune cell infiltration was performed (Figure 7C). The results showed that *ECHDC2*, *FASN*, *NAT8L*, and *AASS* were negatively correlated with aDCs, DCs, iDCs, macrophages, mast cells, neutrophils, T cells, Tgd cells, and Th1 cells. Lollipop plots further illustrated the correlations between each core gene and different immune cells (Figure 7D–G), suggesting that these key MRGs may participate in the occurrence and progression of obesity by regulating the immune microenvironment. Because ssGSEA was performed using bulk adipose tissue transcriptomic data, the observed differences should be interpreted as relative changes in immune-related transcriptional signatures rather than direct measurements of immune cell abundance.

### 3.10. Candidate Small-Molecule Screening and Molecular Docking

Potential drugs targeting hub genes associated with mitochondrial dysfunction were screened using the Enrichr platform, and 10 candidate small-molecule compounds were identified (Table 2 and Supplementary Figure S4). The results showed that most candidate drugs mainly targeted *FASN*, including Rescinnamin, Fisetin, Biochanin A, Quercetin, (−)-Epigallocatechin gallate, Ellagic Acid, Morin, and Redoxal. Genistein was predicted to target both FASN and *NAT8L*, whereas Cianidanol was predicted to target *ECHDC2* and *NAT8L*.

### 3.11. Molecular Docking Between Candidate Drugs and Key Targets

**Figure 8:**
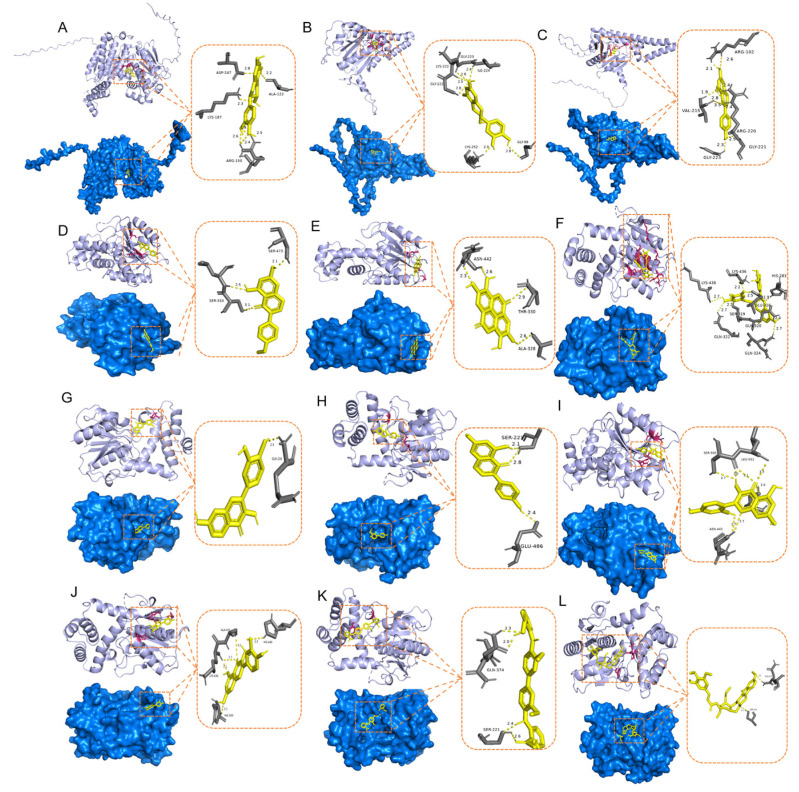
Molecular docking between candidate drugs and key targets. (**A**) ECHDC2–Cianidanol; (**B**) NAT8L–Cianidanol; (**C**) NAT8L–Genistein; (**D**) FASN–Biochanin A; (**E**) FASN–Ellagic Acid; (**F**) FASN–(−)-Epigallocatechin gallate; (**G**) FASN–Fisetin; (**H**) FASN–Genistein; (**I**) FASN–Morin; (**J**) FASN–Quercetin; (**K**) FASN–Redoxal; (**L**) FASN–Rescinnamin.

To evaluate the binding ability between candidate small-molecule drugs and key target proteins, molecular docking analysis was further performed to explore their potential therapeutic value. The results are shown in Figure 8 and Table 3, and additional details on the candidate drugs and their molecular characteristics provided in Supplementary Table S8. Molecular docking was performed as an exploratory and hypothesis-generating analysis for the predicted ligand–target pairs. The predicted AutoDock Vina scores ranged from −7.234 to −8.890 kcal/mol, with the most negative score observed for the Genistein–NAT8L pair. However, because no decoy ligands, unrelated ligands, or experimentally validated benchmark binders were included, these docking scores should be interpreted only as relative computational rankings within the tested set. They do not establish target-specific binding, pharmacological activity, or therapeutic efficacy. The binding free energies of Cianidanol with ECHDC2 and NAT8L were −8.317 kcal/mol and −8.033 kcal/mol, respectively. Several small molecules targeting FASN also showed stable binding affinities, including (−)-Epigallocatechin gallate (−8.313 kcal/mol), Ellagic Acid (−8.265 kcal/mol), and Redoxal (−8.03 kcal/mol).

## 4. Discussion

Obesity is a complex metabolic disease driven by energy metabolic imbalance, adipose tissue dysfunction, and chronic low-grade inflammation. Mitochondria are not only the central sites of fatty acid oxidation, the tricarboxylic acid cycle, and oxidative phosphorylation but also key organelles that link metabolic reprogramming to inflammatory responses. When mitochondrial homeostasis is impaired, adipocytes may exhibit increased oxidative stress, reduced energy expenditure, defective lipid turnover, and aberrant activation of inflammatory signaling, thereby promoting the development and progression of obesity and its related metabolic complications [12,25,26,27]. Based on the integrated analysis of GEO datasets, the present study ultimately identified four core MRGs, namely *ECHDC2*, *FASN*, *NAT8L*, and *AASS*. These four genes showed stable differential expression and high diagnostic performance in both the training and validation datasets, suggesting that they may represent important molecular nodes in mitochondria-related transcriptomic signatures.

GO and KEGG enrichment analyses in this study showed that the differentially expressed MRGs were mainly enriched in carboxylic acid catabolism, small-molecule catabolism, acetyl-CoA/acyl-CoA metabolism, pyruvate metabolism, the TCA cycle, fatty acid biosynthesis, and folate-mediated one-carbon metabolism. These findings suggest that mitochondrial abnormalities associated with obesity are not limited to a single pathway. Previous studies have shown that mitochondrial dysfunction in adipose tissue under obese conditions is often accompanied by decreased membrane potential, impaired fatty acid metabolism, reactive oxygen species accumulation, and abnormal mitochondrial biogenesis, all of which further disrupt the metabolic and endocrine functions of adipocytes [12,25].

Among the four core genes, *FASN* encodes fatty acid synthase, a key rate-limiting enzyme in endogenous de novo fatty acid synthesis [28]. *FASN* expression was significantly downregulated in the OB group, but this downregulation should not be simply interpreted as contradictory to lipid accumulation in obesity. Previous studies have shown that *FASN* expression is associated with visceral fat accumulation, insulin resistance, and metabolic and inflammatory markers such as IL-6, leptin, and RBP4, suggesting that *FASN* is involved in obesity-related metabolic abnormalities, but its role is not simply linear [29]. On the other hand, other studies have found that lipogenic genes, including *FASN/FAS*, show a downward trend in subcutaneous adipose tissue with increasing BMI [30], suggesting that subcutaneous adipose tissue may exhibit decreased lipogenic capacity and adaptive metabolic reprogramming during obesity progression. All datasets included in this study were derived from subcutaneous adipose tissue, which may partly explain the observed decrease in *FASN* expression in the OB group. Furthermore, excessive activation of FASN-mediated fatty acid synthesis may consume NADPH, increase ROS production, and promote adipocyte injury [28,31]. Therefore, *FASN* downregulation may also reflect stress adaptation or metabolic dysfunction in obese adipose tissue rather than simply reduced lipid storage. Another study also showed that FASN deficiency affects autophagosome and lysosomal membrane dynamics in adipocytes [32], indicating that the role of FASN in adipocytes is not limited to triglyceride storage but also involves membrane lipid homeostasis and cellular stress regulation. It should be noted that transcriptomic data cannot directly reflect FASN protein levels, enzymatic activity, or fatty acid synthesis flux, and further protein detection and metabolic flux experiments are required for subsequent validation.

*NAT8L* is another core gene identified in this study. NAT8L catalyzes the production of N-acetylaspartate (NAA) from acetyl-CoA and aspartate and has been linked to adipocyte lipid turnover and mitochondrial metabolic activity in previous experimental studies [33,34]. However, much of the functional evidence for *NAT8L* in adipose metabolism derives from brown adipocytes or adipogenic cell models, whereas the datasets analyzed in the present study were derived from subcutaneous adipose tissue. Therefore, we avoided directly extrapolating brown adipocyte thermogenic mechanisms to the current findings. The observed downregulation of NAT8L in obese subcutaneous adipose tissue should instead be interpreted cautiously as a transcriptomic signal potentially related to altered NAA metabolism, acetyl-CoA utilization, lipid turnover, or mitochondrial metabolic status. Further studies using human subcutaneous adipocytes, including protein-level validation, metabolic flux assays, and functional experiments, are required to determine whether *NAT8L* has a causal role in mitochondria-related transcriptomic signatures in this tissue context.

Compared with *FASN* and *NAT8L*, direct evidence regarding the roles of *AASS* and *ECHDC2* in obesity remains limited; however, their mitochondrial relevance suggests potential biological significance. AASS is a key bifunctional enzyme in the lysine degradation pathway, and lysine degradation through the saccharopine pathway occurs primarily in mitochondria [35]. AASS-related metabolic abnormalities may be accompanied by altered mitochondrial function and impaired energy metabolism [36]. Therefore, the downregulation of *AASS* observed in this study may suggest a potential impairment of amino acid catabolism and mitochondrial substrate metabolism in obese adipose tissue, in addition to lipid metabolic dysregulation. ECHDC2 is a mitochondrial protein, and existing evidence suggests that it may be involved in regulating branched-chain amino acid levels and cellular vulnerability to stress [37]. Although direct evidence linking *ECHDC2* to obesity remains limited, this study found that *ECHDC2* exhibited high AUC values in both the training and validation datasets, suggesting that it may serve as a potential obesity-related mitochondrial biomarker worthy of further validation. Nevertheless, mechanistic interpretations of *AASS* and *ECHDC2* should remain cautious at this stage, and further cellular functional experiments and animal studies are required to clarify their roles in obesity.

In addition to metabolic abnormalities, chronic low-grade inflammation is considered an important driver of obesity progression and obesity-related complications. Obese adipose tissue is not merely an energy storage depot but rather a dynamically remodeled immunometabolic microenvironment, in which immune cell subsets such as macrophages and T cells undergo changes in both abundance and function [27,38]. In the present study, immune infiltration analysis showed that most immune cell types exhibited increased infiltration levels in the OB group. Moreover, the four core MRGs were generally negatively correlated with multiple immune cell types, including aDCs, DCs, iDCs, macrophages, neutrophils, T cells, and Th1 cells. These results suggest that mitochondrial dysfunction may not only reflect metabolic imbalance within adipocytes but may also be closely coupled with remodeling of the obesity-related immune microenvironment. Notably, studies related to *FASN* have indicated that fatty acid synthesis metabolism can affect immune cell activation and functional status [39]. Therefore, reduced expression of *ECHDC2*, *FASN*, *NAT8L*, and *AASS* may contribute to, or at least reflect, inflammatory activation and abnormal immune cell infiltration in adipose tissue by affecting lipid synthesis, oxidative stress, and metabolic substrate supply.

Among the predicted transcription factors, several candidates have potential biological relevance to adipose tissue remodeling. NFIC has been reported to regulate the balance between adipogenic and osteogenic differentiation through canonical Wnt signaling, suggesting a possible role in adipose tissue remodeling and metabolic regulation [40]. Direct evidence linking PRRX2 to obesity remains limited; therefore, its potential involvement should be interpreted with caution. However, the related paired-homeobox transcription factor PRRX1 has been shown to suppress adipogenic differentiation via TGF-β signaling and has been associated with adipose tissue dysfunction in obesity [41,42]. FOXC1 may also be related to adipogenic regulation, as Foxc1 deficiency promotes adipocyte accumulation in mesenchymal niche cells and affects adipocyte differentiation [43,44], although its precise role in adipose tissue metabolism remains unclear. In addition, TFAP2A has been implicated in adipogenesis-related transcriptional regulation [45,46], including evidence suggesting that it may act as an inhibitor of adipogenic differentiation. Nevertheless, because these TFs were predicted using database-based regulatory network analysis, their direct regulatory effects on *ECHDC2*, *FASN*, *NAT8L*, and *AASS* should be regarded as hypothesis-generating and require further experimental validation.

Furthermore, the miRNA–mRNA–TF regulatory network constructed in this study suggested that the core MRGs may be regulated at multiple transcriptional and post-transcriptional levels. Several predicted miRNAs in the regulatory network have previously been linked to adipose biology. For example, miR-16-5p has been shown to promote 3T3-L1 adipocyte differentiation and lipid droplet accumulation by targeting EPT1 [47], suggesting a role in adipogenesis. miR-17-5p was reported to be increased in mouse white adipose tissue after high-fat diet feeding and to participate in adipogenic regulation [48], although its function may be context- and species-dependent. miR-484 has been implicated in preadipocyte proliferation and adipogenic differentiation [49]. In addition, the miR-193b-365 cluster has been identified as an important regulator of brown fat development [50], although this evidence should be extrapolated cautiously to the subcutaneous adipose tissue context of the present study. In contrast, direct evidence linking miR-615-3p to obesity or human adipose tissue biology remains limited; available studies mainly suggest its involvement in cellular differentiation or lipid-stress-related pathways [51,52]. Therefore, miR-16-5p, miR-17-5p, miR-484, and miR-193b-related regulation may represent higher-priority candidates for future validation, whereas miR-615-3p should be interpreted as a lower-confidence predicted regulator. The sparsity of the regulatory network also indicates that the robustness of individual TF– or miRNA–MRG interactions may be limited. Nodes regulating multiple core MRGs may represent higher-priority candidates for future validation, whereas singly connected nodes should be interpreted with caution. Therefore, the predicted regulatory network provides a preliminary framework for identifying potential upstream regulators, but experimental approaches such as qRT-PCR, luciferase reporter assays, chromatin immunoprecipitation, and gain- or loss-of-function studies are required to confirm these regulatory relationships.

Several recent bioinformatics studies have investigated obesity-related biomarkers using transcriptomic datasets and machine learning approaches. For example, previous studies identified metabolism-related genes such as *STOX1* and *NWD2* [53] or adipose tissue-associated obesity genes such as *EGR2*, *GREM1*, and *NPY1R* [54]. These studies support the value of transcriptomic and machine learning strategies for identifying obesity-associated molecular signatures. However, most of them focused on general metabolic or adipose tissue-related biomarkers rather than specifically prioritizing mitochondria-related genes. Compared with these studies, the present work specifically integrated obesity-related DEGs with the MitoCarta3.0 mitochondria-related gene set, thereby focusing on mitochondrial dysfunction as a mechanistic framework. In addition, we combined candidate gene prioritization with immune infiltration analysis, regulatory network prediction, drug screening, and molecular docking. Nevertheless, consistent with previous computational studies, our findings should be interpreted as hypothesis-generating and require further validation at the protein, metabolic flux, cellular, and clinical cohort levels.

The candidate small molecules identified in this study, including quercetin, (−)-Epigallocatechin gallate, genistein, and biochanin A, may exert potential anti-inflammatory and antioxidant effects, improve lipid metabolism, and alleviate obesity-related metabolic abnormalities [55,56,57,58]. The molecular docking results further suggested that these compounds have favorable binding potential with *FASN* or *NAT8L*, providing preliminary evidence for subsequent targeted intervention studies. However, molecular docking only reflects a static binding tendency and is insufficient to confirm actual in vivo efficacy. Therefore, these molecules should currently be regarded as candidate lead compounds for further experimental screening rather than confirmed therapeutic agents.

## 5. Conclusions

In conclusion, this study identified *ECHDC2*, *FASN*, *NAT8L*, and *AASS* as prioritized mitochondria-related candidate genes. Beyond confirming the involvement of mitochondrial metabolic disturbance in obesity, our findings highlight a coordinated transcriptomic signature related to lipid metabolism, amino acid catabolism and immune microenvironment remodeling. Further protein-level, metabolic flux, cellular, and clinical cohort studies are required to confirm their functional roles and translational relevance. However, these genes should currently be regarded as hypothesis-generating candidates rather than experimentally validated diagnostic biomarkers or therapeutic targets. Further validation at the mRNA, protein, metabolic, mitochondrial functional, cellular, and clinical cohort levels is required.

## 6. Limitations

This study has several limitations. First, this study was based on public transcriptomic datasets with a relatively limited sample size, which may increase the risk of model overfitting, particularly in LASSO and RF analyses. Although batch effect correction and external validation were performed, the high AUC values observed in this study, including those approaching or equal to 1.0, should be interpreted with caution and should not be considered established evidence of clinical diagnostic performance. Second, all samples were derived from subcutaneous adipose tissue; therefore, the findings may not be directly generalizable to visceral adipose tissue, brown adipose tissue, or other metabolically active tissues. Third, the integration of different GEO datasets and Affymetrix microarray platforms may still introduce residual platform-related bias or batch-correction artifacts despite correction procedures. Fourth, the core mitochondria-related genes were prioritized from the same set of differentially expressed MRGs through differential expression analysis, PPI network analysis, and machine learning, which may introduce potential circularity. Thus, these genes should be regarded as prioritized candidate biomarkers rather than independently validated targets. Fifth, this study lacks experimental validation at the protein, metabolic, mitochondrial functional, and gene functional levels. In addition, immune infiltration was inferred from bulk adipose tissue transcriptomic data. Therefore, the immune-related findings should be considered exploratory and require validation using cell-resolution approaches. Finally, regulatory network construction, candidate drug screening, and molecular docking were based on computational predictions and reflect potential associations rather than causal relationships. In particular, the predicted polyphenol compounds have broad multi-target effects, and direct evidence supporting their specific effects on *ECHDC2*, *FASN*, *NAT8L*, or AASS is lacking. In addition, the molecular docking analysis did not include decoy ligands, unrelated compound controls, or experimentally validated reference binders. Moreover, the molecular docking analyses based on AlphaFold-predicted structures should be regarded as exploratory and hypothesis-generating. For proteins lacking experimentally resolved ligand-bound structures or experimentally validated binding sites, the docking results should be interpreted with particular caution. Therefore, the candidate drugs identified here should be interpreted as preliminary computational clues rather than specific therapeutic evidence targeting the core MRGs.

## Figures and Tables

**Table 1:** Basic Information and GEO Characteristics of the Included Datasets.

GEO Accession	Platform	Tissue Sample	Gene Expression Microarray Platform	Submission Date	Country	OB Samples	NW Samples	Total Samples
GSE94752	GPL11532	Subcutaneous adipose tissue	Affymetrix Human Gene 1.1 ST Array	2017	Sweden	21	9	30
GSE55200	GPL17692	Subcutaneous adipose tissue	Affymetrix Human Gene 2.1 ST Array	2014	Canada	8	7	15
GSE151839	GPL570	Subcutaneous adipose tissue	Affymetrix Human Genome U133 Plus 2.0 Array	2020	USA	10	10	20

**Table 2:** Candidate drugs and their molecular characteristics.

Name	Molecular Formula	*p*-Value	Structure	Related Genes
Rescinnamin	C_35_H_42_N_2_O_9_	2.60 × 10^3^	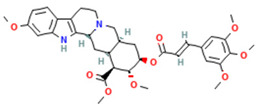	*FASN*
Fisetin	C_15_H_10_O_6_	4.19 × 10^3^	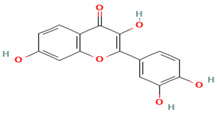	*FASN*
Biochanin A	C_16_H_12_O_5_	4.99 × 10^3^	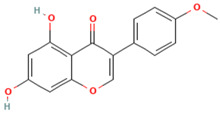	*FASN*
Quercetin	C_15_H_10_O_7_	6.78 × 10^3^	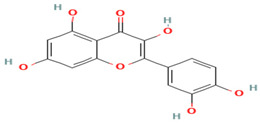	*FASN*
(−)-Epigallocatechin gallate	C_22_H_18_O_11_	5.99 × 10^3^	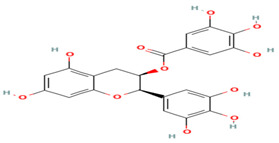	*FASN*
Ellagic Acid	C_14_H_6_O_8_	1.53 × 10^2^	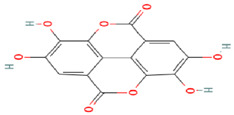	*FASN*
Morin	C_15_H_10_O_7_	5.39 × 10^3^	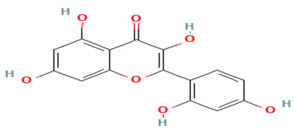	*FASN*
Genistein	C15H10O5	2.09 × 10^2^	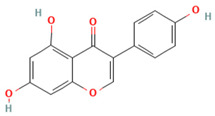	*FASN*; *NAT8L*
Redoxal	C_28_H_24_N_2_O_6_	7.58 × 10^3^	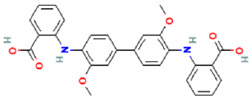	*FASN*
Cianidanol	C_15_H_14_O_6_	1.07 × 10^2^	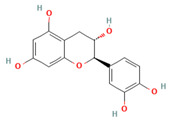	*ECHDC2*; *NAT8L*

**Table 3:** Binding free energies of candidate small molecules with target proteins.

Small-Molecule Ligand	Target Receptor Protein	Binding Free Energy (kcal/mol)
Cianidanol	ECHDC2	−8.317
Cianidanol	NAT8L	−8.033
Genistein	NAT8L	−8.89
Biochanin A	FASN	−7.29
Ellagic Acid	FASN	−8.265
(−)-Epigallocatechin gallate	FASN	−8.313
Fisetin	FASN	−7.835
Genistein	FASN	−7.456
Morin	FASN	−7.63
Quercetin	FASN	−7.456
Redoxal	FASN	−8.03
Rescinnamin	FASN	−7.234

## Data Availability

The data analyzed in this study were obtained from publicly available resources. Supplementary information and related materials supporting the findings of this study are available from the corresponding authors upon reasonable request.

## Supplementary Materials

The following supporting information can be downloaded at https://www.mdpi.com/article/10.3390/metabo16060419/s1, Figure S1: PCA analysis of the training datasets before and after batch-effect correction; Figure S2: Correlation heatmaps of the four core genes in the training and validation datasets; Figure S3: Simplified miRNA-MRG-TF regulatory subnetwork of the four core mitochondria-related genes; Figure S4: Drug–target interaction network of candidate compounds and core MRGs; Table S1: Clinical and metabolic characteristics of the included GEO datasets; Table S2: Housekeeping gene expression consistency before and after batch-effect correction; Table S3: Structural information of target proteins used or considered in molecular docking analysis; Table S4: Numbers of differentially expressed genes identified in the individual datasets and the batch-corrected combined training cohort; Table S5: Top-ranked genes identified by five CytoHubba algorithms in the PPI network; Table S6: Rankings and importance scores of candidate genes across machine learning algorithms; Table S7: Differential expression of core mitochondria-related genes in the training and validation cohorts; Table S8: Candidate drugs and their molecular characteristics. Reported IC_50_ information for quercetin and ellagic acid was obtained from Zhao et al. [59] and Wu et al. [60], respectively.
