# Supplementary material for: Identification and Transcriptomic Analysis of Mitochondria-Related Gene Signatures in Obesity
Source: Metabolites. 2026 Jun 15;16(6):419. doi: 10.3390/metabo16060419 (PMC13303004; doi:10.3390/metabo16060419)
Supplement: Supplementary file 1 [file metabolites-16-00419-s001.zip › metabolites-4321606-supplementary.pdf]

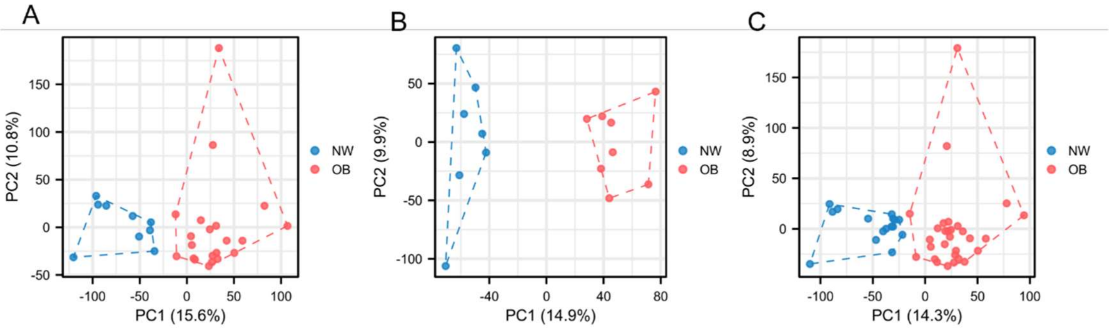

Supplementary Figure S1. PCA analysis of the training datasets before and after batch-effect correction. (A) PCA plot of GSE94752. (B) PCA plot of GSE55200. (C) PCA plot of the merged training cohort after batch-effect correction using limma, removeBatchEffect. Samples are colored according to group status. OB, obesity group; NW, normal-weight control group.

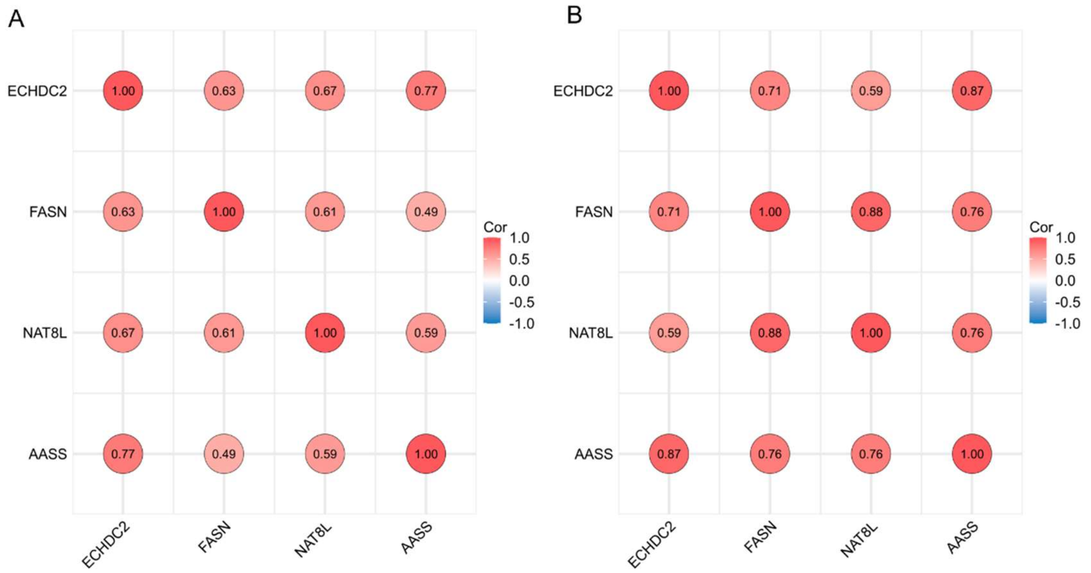

Supplementary Figure S2. Correlation heatmaps of the four core genes in the training and validation datasets. (A) Correlation heatmap of ECHDC2, FASN, NAT8L, and AASS in the training dataset. (B) Correlation heatmap of ECHDC2, FASN, NAT8L, and AASS in the validation dataset. The numbers in each cell indicate the pairwise correlation coefficients.

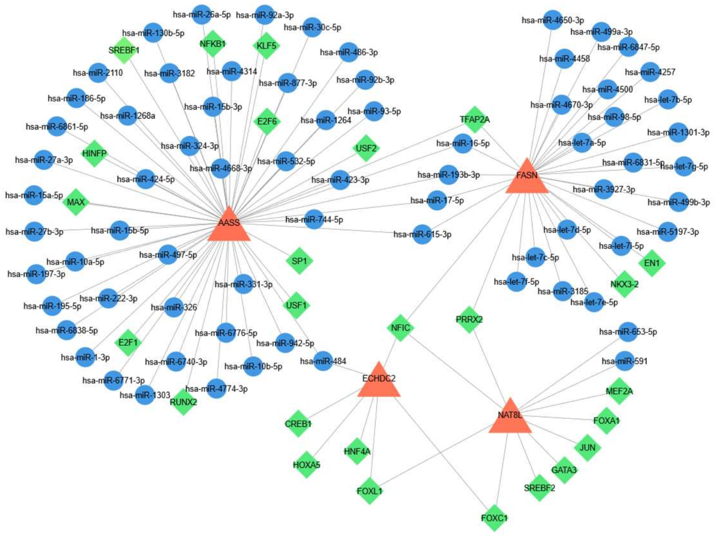

Supplementary Figure S3. Simplified miRNA-MRG-TF regulatory subnetwork of the four core mitochondria-related genes.

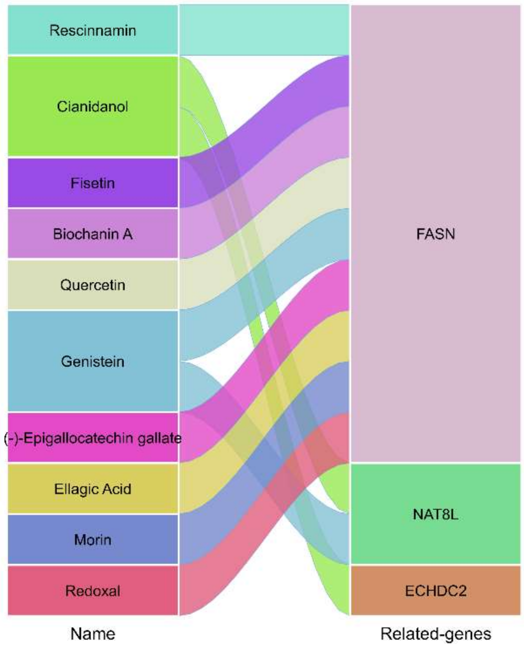

Supplementary Figure S4. Drug-target interaction network of candidate compounds and core MRGs.

Supplementary Table S1. Clinical and metabolic characteristics of the included GEO datasets

Dataset	BMI in OB group (kg/m²)	BMI in NW grou (kg/m²)	Age in OB group (years)	Age in NW group (years)	Sex distribution	Metabolic criteria / sample description
GSE94752	41 ± 5	23 ± 1	36.24±7.76	37.44 ±10.63	Female	obese insulin-sensitive subjects and lean controls
GSE55200	≥28 for males; ≥24 for females	<28 for males; <24 for females	35-70	35-70	Female and male	Lean healthy controls and metabolically healthy obese subjects
GSE151839	35–50	18.5–26.9	40-70	40-70	female	obese subjects and normal-weight controls

Supplementary Table S2. Housekeeping gene expression consistency before and after batch-effect correction.

Analysis	Pearson *r*	Pearson P	Spearman ρ	Housekeeping gene score *P* value
Before correction	0.178	0.774	0.400	6.47×10^{-8}
After correction	0.999999	4.61×10^{-10}	1.000	0.857

Supplementary Table S3. Structural information of target proteins used or considered in molecular docking analysis

Target protein	Accession / model ID	Structure source	Structure type	Use in docking	Mean pLDDT	Ramachandran plot results, favored/allowed/outliers (%)
ECHDC2	Q86YB7	AlphaFold DB	Predicted model	Yes	91.09	94.31 / 3.97 / 1.72
FASN	8VLP	PDB	Experimental cryo-EM structure	Yes	N/A	N/A
NAT8L	Q8N9F0	AlphaFold DB	Predicted model	Yes	79.14	97.00 / 1.67 / 1.33
AASS	5L78	PDB	Experimental crystal structure, SDH domain	No	N/A	N/A

Supplementary Table S4. Numbers of differentially expressed genes identified in the individual datasets and the batch-corrected combined training cohort.

Dataset	Upregulated DEGs	Downregulated DEGs	Total DEGs
GSE94752	678	273	951
GSE55200	251	148	399
Combined training cohort after batch correction	386	141	527

Supplementary Table S5. Top-ranked genes identified by five CytoHubba algorithms in the PPI network.

Gene	MNC rank	Degree rank	EPC rank	DMNC rank	MCC rank	Included in final PPI intersection
PC	1	1	1	5	1	Yes
FASN	1	2	2	5	2	Yes
ALAS2	5	5	7	4	6	Yes
MOCS1	6	6	10	6	8	Yes
AASS	2	3	4	3	3	Yes
NAT8L	6	6	9	6	8	Yes
ALDH1L1	2	3	3	3	3	Yes
ECHDC2	3	3	5	2	4	Yes
LDHD	4	4	6	1	5	Yes
BCL2A1	6	5	8	6	7	Yes

Supplementary Table S6. Rankings and importance scores of candidate genes across machine learning algorithms

Gene Symbol	RF	LASSO	Selected by RF	Selected by LASSO	In Final Intersection
BOK	8.881	-0.244	Yes	Yes	Yes
NIPSNAP3B	12.771	-3.814	Yes	Yes	Yes
LDHD	2.896	0.000	No	No	No
ALDH1L1	7.581	0.000	Yes	No	No
PC	7.466	0.000	Yes	No	No
ECHDC2	9.868	-3.176	Yes	Yes	Yes
NAT8L	8.050	-4.227	Yes	Yes	Yes
PXMP2	4.407	0.000	Yes	No	No

Gene Symbol	RF	LASSO	Selected by RF	Selected by LASSO	In Final Intersection
AASS	9.678	-0.076	Yes	Yes	Yes
MOCS1	4.248	0.000	Yes	No	No
BCL2A1	4.125	0.000	Yes	No	No
FASN	4.248	-0.524	Yes	Yes	Yes
COX14	5.210	-2.034	Yes	Yes	Yes
PLD6	4.345	-2.185	Yes	Yes	Yes
ALAS2	3.017	0.982	No	Yes	No

RF: Random Forest MeanDecreaseAccuracy

Supplementary Table S7. Differential expression of core mitochondria-related genes in the training and validation cohorts

Cohort	Gene symbol	Expression in OB, mean ± SD	Expression in NW, mean ± SD	log_2FC	Adjusted P value
Training cohort	ECHDC2	6.802 ± 0.282	7.466 ± 0.295	-0.664	1.23×10^{-7}
Training cohort	FASN	10.227 ± 0.621	11.022 ± 0.409	-0.795	2.74×10^{-4}
Training cohort	NAT8L	7.222 ± 0.334	7.770 ± 0.164	-0.549	3.95×10^{-6}
Training cohort	AASS	7.346 ± 0.329	8.180 ± 0.334	-0.834	2.10×10^{-8}
Validation cohort	ECHDC2	10.380 ± 0.100	10.919 ± 0.185	-0.539	2.64×10^{-4}
Validation cohort	FASN	7.011 ± 0.413	7.738±0.269	-0.727	3.86×10^{-3}
Validation cohort	NAT8L	7.882 ± 0.544	8.527 ± 0.268	-0.645	2.27×10^{-2}
Validation cohort	AASS	7.611± 0.129	8.532 ± 0.486	-0.921	1.02×10^{-3}

Supplementary Table S8. Candidate drugs and their molecular characteristics

Name	Related-genes	Reported IC_{50}	Evidence type	Reference
Rescinnamin	FASN	Not reported	No reliable FASN-specific	-

			IC_{50} found	
Fisetin	FASN	Not reported	Non-human FAS assay	-
Biochanin A	FASN	Not reported	No reliable FASN-specific IC_{50} found	-
Quercetin	FASN	4.29 ± 0.34 μM	FASN activity inhibition	Zhao et al., 2014 [59]
(−)-Epigallocatechin gallate	FASN	Not reported	Non-human FASN/FAS-related assay	-
Ellagic Acid	FASN	1.31 μg/mL, approximately 4.34 μM	FAS activity inhibition	Wu et al., 2013 [60]
Morin	FASN	Not reported	Non-human FAS assay	-
Genistein	FASN; NAT8L	Not reported	No reliable target-specific IC_{50} found	-
Redoxal	FASN	Not reported	No reliable FASN-specific IC_{50} found	-
Cianidanol	ECHDC2; NAT8L	Not reported	No reliable target-specific IC_{50} found	-
